# Rhabdomyosarcome cranio-facial de l’adulte: un cas de mauvais pronostic

**DOI:** 10.11604/pamj.2018.30.3.14690

**Published:** 2018-05-03

**Authors:** Adil Arrob, Mohammed Kamal Fiqhi, Abibou N’diaye, Karim El Khatib, Abdeljalil Abouchadi

**Affiliations:** 1 Service de Chirurgie Plastique, Maxillo-faciale et Stomatologie, Hôpital Militaire Avicenne, Marrakech, Maroc; 2 Service de Chirurgie Plastique, Maxillo-faciale et Stomatologie, Hôpital Militaire d’Instruction Mohammed V, Rabat, Maroc

**Keywords:** Rhabdomyosarcome, cranio-facial, adulte, prognostic, Rhabdomyosarcoma, craniofacial, adult, prognosis

## Abstract

Le rhabdomyosarcome est la tumeur mésenchymateuse la plus fréquente chez l'enfant et l'adolescent. Il représente 60 à 70% des tumeurs mésenchymateuses et environ 5% de l'ensemble des tumeurs solides à ces âges. Près de la moitié des rhabdomyosarcomes surviennent au niveau de la tête et du cou. Nous rapportons le cas d'un rhabdomyosarcome agressif à localisation temporo-pariétale. Chez un jeune adolescent de 20 ans avec une forme histologique nouvelle.

## Abstract (en)

Rhabdomyosarcoma is the more frequent mesenchymal tumor in children and in adolescents. It accounts for 60 -70% of mesenchymal tumors and approximately 5% of all solid tumors occurring at these ages. Almost half of rhabdomyosarcomas occur in the head and the neck. We report the case of a 20-year old patient with a new histological, aggressive temporo-parietal rhabdomyosarcoma.

## Introduction

Le rhabdomyosarcome(RMS) est une tumeur maligne d'origine mésenchymateuse comportant une différenciation musculaire striée plus ou moins marquée, d'étiologie inconnue [1]. C'est une tumeur du nourrisson ou de l'enfant en bas âge, rare chez l'adulte. Les localisations céphaliques sont parmi les plus fréquentes avec une fréquence de 41% de l'ensemble des rhabdomyosarcomes [2]. C'est une tumeur de haut degré de malignité qui se distingue des autres sarcomes par son agressivité locorégionale, son évolution métastatique et son pronostic défavorable quel que soit le traitement entreprit. Le diagnostic n'est posé qu'à l'examen histologique. La prise en charge est multidisciplinaire associant chirurgie, radiothérapie et chimiothérapie.

## Patient et observation

**Figure 1:**
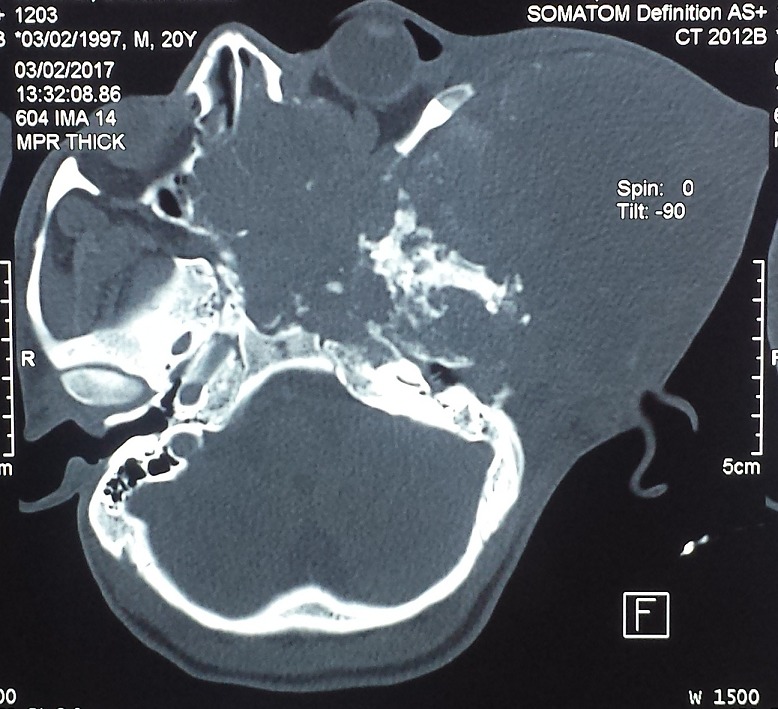
Aspect à la tomodensitométrie, coupe axiale montrant un volumineux processustumoral agressif, sphéno-temporo-pariéto-orbitaire, responsable d’une ostéolyse diffuse

**Figure 2:**
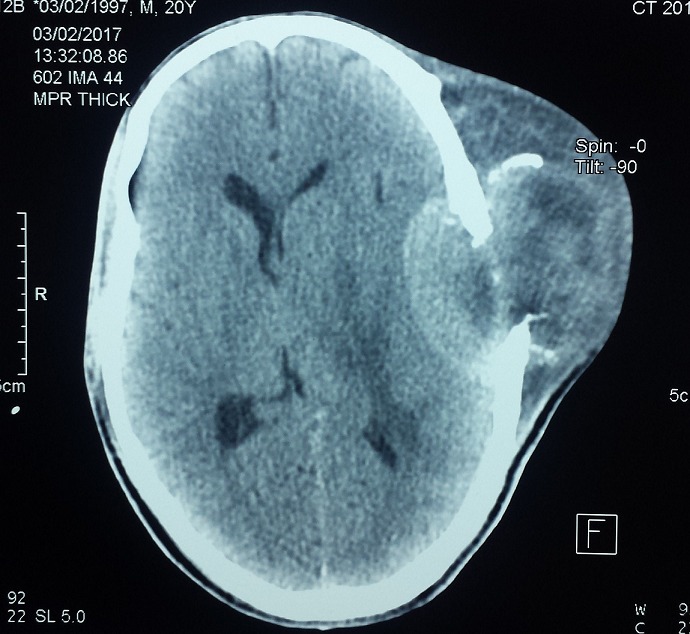
Aspect à la tomodensitométrie cérébrale, coupe pariétale montrant l’ostéolyse et extension endocrânienne de la tumeur

**Figure 3:**
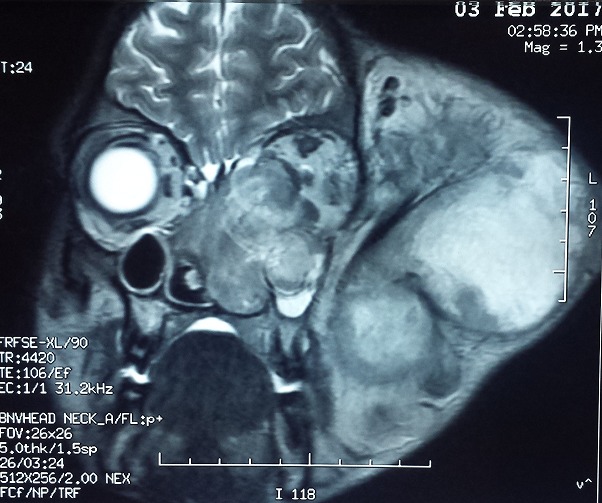
Aspect en imagerie par résonance magnétique coupe coronale : l’envahissement tumorale de l’hémiface gauche

Notre travail concerne un patient âgé de 20 ans, du Sud Soudan, adressé pour la prise en charge d'une énorme tumeur de la région temporale gauche. L'anamnèse ne trouve pas d'antécédents pathologiques connus, le patient n'a jamais été opéré, pas de notion de traumatisme facial. Le début de la symptomatologie remonte à 6 mois par l'apparition d'un nodule sous-cutané de la région temporale gauche qui a rapidement augmenté de taille avec une baisse de l'acuité visuelle de l'œil gauche, une hypoacousie gauche et un écoulement muqueux nasal gauche, le tout évoluant dans un contexte de conservation de l'état général. L'examen général trouve un patient en bon état général, amaigri pesant 55 kg pour une taille de 1,75 m. L'examen clinique trouve une tumeur géante sous-cutanée de l'hémiface gauche centrée sur la région temporo-pariétale de dimension 11 x 12 cm, de consistance tissulaire dure en périphérie et fluctuante au centre, n'infiltrant pas la peau qui est saine, sans signe inflammatoire, indolore à la palpation. On note une exophtalmie gauche non axiale, pas de diplopie, pas de déficit moteur; Une hypoesthésie sous orbitaire (V2) gauche, une obstruction narinaire homolatérale et une limitation de l'ouverture buccal à 25 mm ; on ne trouve pas de lésion endobuccale. L´examen des aires ganglionnaires cervicales ne met pas en évidence d´adénopathies palpables. Le reste de l'examen somatique est sans particularité. Une tomodensitométrie crânio-faciale (Figure 1) a montré la présence d'un volumineux processus tumoral agressif, sphéno-temporo-pariéto-orbitaire, responsable d'une ostéolyse mitée diffuse du squelette de l'hémiface et de la base du crâne gauche avec ostéolyse pariéto-temporale et extension endocrânienne(Figure 2); ce processus tissulaire contenant des calcifications et de volumineuses logettes de nécrose, mesurant (15 cm x 9 cm x 14,5 cm), on note la présence d'un œdème cérébral périphérique pariéto-temporal gauche avec un effet de masse sur le ventricule latéral. L'imagerie par résonance magnétique (Figure 3) donne plus de précision sur ce processus tumoral qui infiltre en dehors, les parties molles temporales, jugales (le masséter) et le tissu cutané, en avant, il infiltre l'orbite, englobe le nerf optique, les muscles oculomoteurs avec exophtalmie grade 3, le sinus sphénoïdal, les cellules ethmoïdales, les fosses nasales et le sinus maxillaire gauche. En bas il infiltre la fosse infra temporale et les muscles ptérygoïdiens.

**Figure 4:**
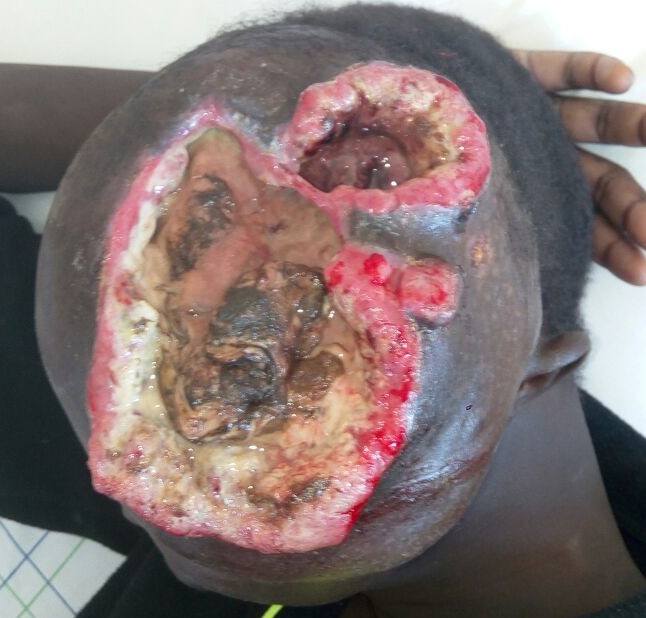
Image du patient, vue de profil droit, montrant l’extension et la nécrose tumorale

Le patient a bénéficié d'une biopsie chirurgicale sous neuroleptanalgésie vu le risque d'engagement au cours d'une anesthésie générale. L'examen anatomopathologique a montré, à la coupe, un derme totalement occupé par une lésion tumorale d'aspect blanchâtre et comportant des zones de remaniements myxoides. L'étude microscopique a objectivé une prolifération sarcomateuse peu différenciée de haut grade. L'étude immunohistochimie a montré que les cellules tumorales expriment de façon focale et intense la CK ae1-ae3, le BCL2 , la desmine et l'actine du muscle lisse et n'expriment pas la PS100, le CD 34, le CD 31, la myogénine, le CD99, l'HM45 el le Melan A, ce qui a conclu à un sarcome épitheloide de type proximal (grade 3 de la Fédération nationale des centres de lutte contre le cancer (FNCLCC)) [3]. Le bilan d'extension comportant une tomodensitométrie thoraco-abdomino-pelvienne et une scintigraphie osseuse n'ont révélé aucune localisation secondaire. Après réunion de concertation pluridisciplinaire (RCP) la tumeur a été jugée non accessible à un traitement chirurgical d'emblée et une chimiothérapie néoadjuvante a été proposée après un bilan de tolérance complet. Le patient a reçu la première cure de chimiothérapie selon le Protocole AI (Adriablastine-Ifosfamide) avec une tolérance correcte mais sans réel bénéfice clinique. La relecture des lames réalisée dans un centre spécialisé a permis d'affiner le diagnostic en retenant le diagnostic de rhabdomyosarcome de sous-type SAI. Le protocole de chimiothérapie a été changé suite à cette relecture et le patient a reçu une cure selon le protocole VAC (Vincristine, Actinomycine, Cyclophosphamide). L'évolution est marquée par une stabilité de la masse aussi bien clinique que sur la tomodensitométrie de réévaluation. Après cette chimiothérapie néoadjuvante, et devant l'inopérabilité chirurgicale, la RCP a proposé une radiothérapie palliative avec un traitement médicale par Pazopanib. L'évolution clinique a été marquée par un affaiblissement progressif du patient, par l'extension et la nécrose tumorale (Figure 4), la douleur était supportable, le patient a présenté des épisodes d´épistaxis qui ont nécessité un méchage, le décès est survenu 6 mois après l´admission du patient.

## Discussion

Le rhabdomyosarcome est une tumeur très agressive se présentant comme une prolifération de cellules peu différenciées rondes ou fusiformes avec une ligne de différenciation musculaire striée. L'immunohistochimie est d'une aide précieuse, recherchant un marquage des rhabdomyoblastes par myosine, actine, desmine, myoglobine et Myo-D [4]. On distingue classiquement trois sous types histologiques de RMS: 1) le RMS de type embryonnaire qui représente plus des deux tiers des RMS de l'enfant. Deux sous-types ont été décrits qui seraient de meilleur pronostic: botryoïde, forme bourgeonnante en grappes (surtout fréquente au niveau du cavum chez l'enfant) et à cellules fusiformes (surtout para-testiculaire); 2) le RMS alvéolaire qui représente 20% des histologies chez l'enfant, mais ce taux augmente dans les séries de rhabdomyosarcome de l'adulte. Histologiquement on retrouve d'épais septa fibreux et une perte de la cohésion des cellules, (alvéoles). La prolifération souvent homogène de cellules rondes à cytoplasme clair peut poser des problèmes de diagnostic différentiel avec d'autres tumeurs à petites cellules rondes bleues comme les lymphomes.

Au niveau tête et cou, l'histologie alvéolaire serait surtout fréquente dans les localisations naso-sinusiennes, le RMS pléomorphe est une forme de l'adulte dont la fréquence augmente avec l'âge [5] et qui semble rare voire inexistante au niveau cervico-facial. L'examen anatomopathologique dans le cas présenté montre une prolifération, d'une part de cellules fusiformes disposés de manière fasciculée et d'autre part de petites cellules rondes disposées en plage sans architecture. Les cellules présentent un noyau rond ou allongé, monomorphe, régulier mais chromatique et avec la présence de nombreuses mitoses. L'étude immunohistochimique montre une positivité diffuse à la pancytokératine (AE1/AE3), à l'anti desmine et l'anti Ini1, une positivité focal à la myogénine et une négativité totale à l'ALK1 avec un Ki67 à 80%. L'aspect morphologique et immunohistochimique en faveur d'un rhabdomyosarcome SAI de très haut grade de malignité et d'une forme très particulière associant un phénotype épithéliale et musculaire strié correspond très vraisemblablement à un nouveau type de rhabdomyosarcome avec un transcrit rare. L'étude biologie moléculaire montre l'absence de détection de mutation dans l'exon 1 du gène MYOD1. Le RMS est le sarcome des tissus mous (STM) le plus fréquent chez l'enfant, la localisation tête et cou représentant environ 40% des cas. Chez l'adulte cette histologie est beaucoup plus rare (moins de 3% de l'ensemble des STM) mais est tout de même l'histologie la plus fréquents de sarcome TC, notamment au niveau du massif facial. Les RMS sont préférentiellement localisés aux cavités naso-sinusiennes et à l'espace masticateur. On distingue les RMS de localisation para méningée et non para méningée (cou, orbite) rares chez l'adulte. Les premiers exposent à un taux plus élevé d'adénopathies et de métastases initiales et sont moins accessibles chirurgicalement du fait des possibilités d'envahissement endocrânien. Les RMS de l'adulte ont une lymphophilie particulière (15 à 50%) notamment dans les localisations para méningées (> 40 %) d'atteinte ganglionnaire au diagnostic [6].

Le bilan doit donc être exhaustif comportant un bilan biologique, un scanner thoraco-abdominal, une échographie hépatique, une scintigraphie osseuse, la tomographie à émission de positons au fluorodésoxyglucose(TEP-FDG) [7], une biopsie médullaire et éventuellement une ponction lombaire dans les localisations paraméningées. La classification clinique des RMS de l'adulte la plus utilisée, notamment pour toutes les formes alvéolaires et embryonnaires, est celle issue des groupes coopératifs pédiatriques (classification de l'Intergroup Rhabdomyosarcoma Study (IRS)) [8]. Elle tient compte de la localisation, de la taille, du caractère paraméningé et de l'extension à distance ainsi que de la résection chirurgicale. Il apparaît souhaitable d'utiliser cette classification pour les RMS de la tête et du cou compte tenu des implications pronostiques spécifiques de cette histologie. De façon générale, compte tenu du risque métastatique majeur, les RMS de l'adulte relèvent d'une chimiothérapie néoadjuvante associée à un traitement locorégional à l'instar de ce qui est réalisé dans les cas pédiatriques. Si le bénéfice de ce traitement n'a pas été mis en évidence de façon significative chez l'adulte, contrairement à l'enfant, c'est probablement dû aux faibles effectifs des séries d'adultes et à l'hétérogénéité des traitements proposés. Les protocoles de chimiothérapie utilisés chez l'adulte tendent cependant à devenir similaires aux protocoles utilisés en pédiatrie compte tenu des excellents résultats thérapeutiques obtenus dans cette population en terme de survie [9]. Les protocoles de chimiothérapie associent le plus souvent Vincristine, Actinomycine, Cyclophosphamide, Etoposide, Ifosfamide, Doxorubicine. La tolérance de ces protocoles très lourds constitue un facteur limitant chez l'adulte au-delà de 25 ans. Après chimiothérapie néo-adjuvante de 12 semaines, le traitement chirurgical est réalisé en fonction de l'opérabilité. Le traitement des aires ganglionnaires est associé à celui du site tumoral. La chirurgie de ces tumeurs souvent localement étendues est complexe, mais ce geste avant la RT a pour objectif d'améliorer la probabilité de contrôle local. Il faut cependant nuancer la place de la chirurgie dans le traitement des localisations paraméningées, qui sont souvent localement étendues sur le plan locorégional et exposent rapidement à une dissémination métastatique. Les rhabdomyosarcomes étant des tumeurs très radiosensibles, la radiothérapie fait également partie intégrante du traitement. La dose utilisée en pédiatrie est de 50,4Gy pour les rhabdomyosarcomes embryonnaires et alvéolaires [9]. Chez l'adulte, compte tenu d'un taux supérieur d'histologies non embryonnaires, l'augmentation de la dose aurait un intérêt et serait facilitée par les techniques conformationnelles. L'irradiation concerne le site tumoral et les aires ganglionnaires. Elle est habituellement réalisée en postopératoire. Cependant, dans les formes à haut risque métastatique, pour lesquelles la chirurgie risque d'être mutilante, un traitement à visée curative par radiochimiothérapie exclusive doit être discuté, notamment si la chimiothérapie a entraîné une régression tumorale majeure.

## Conclusion

La prise en charge des rhabdomyosarcomes est multidisciplinaire, comportant polychimiothérapie, chirurgie et radiothérapie externe. Malgré l´amélioration de la prise en charge thérapeutique, le pronostic des rhabdomyosarcomes tête et cou de l'adulte reste très sombre compte tenu d'un stade souvent initialement avancé de la maladie, de l'inopérabilité fréquente en cas d'extensions basicrâniennes et du haut potentiel métastatique.

## Conflits d’intérêts

Les auteurs ne déclarent aucun conflit d´intérêts.

## References

[1] Danden A, Hartmann O, Vassal G, Oberlin O (2003). Tumeurs mésenchymateuses malignes ou sarcomes des tissus mous.

[2] Gil Z, Patel S G, Singh B, Cantu G, Fliss D M, Kowalski L P, Shah J P (2007). Analysis of prognostic factors in 146 patients with anterior skull base sarcoma: an international collaborative study. Cancer.

[3] Trojani M, Contesso G, Coindre JM, Rouesse J, Bui NB, De Mascarel A, Lagarde C (1984). Soft-tissue sarcomas of adults; study of pathological prognostic variables and definition of a histopathological grading system. International Journal of Cancer.

[4] Paulino AC, Okcu MF (2008). Rhabdomyosarcoma. Current problems in cancer.

[5] Little DJ, Ballo MT, Zagars GK, Pisters PW, Patel SR, El-Naggar AK, Benjamin RS (2002). Adult rhabdomyosarcoma. Cancer.

[6] Nayar RC, Prudhomme F, Parise O, Gandia D, Luboinski B, Schwaab G (1993). Rhabdomyosarcoma of the head and neck in adults: a study of 26 patients. The Laryngoscope.

[7] Bastiaannet E, Groen H, Jager PL, Cobben DCP, Van Der Graaf WTA, Vaalburg W, Hoekstra HJ (2004). The value of FDG-PET in the detection, grading and response to therapy of soft tissue and bone sarcomas; a systematic review and meta-analysis. Cancer treatment reviews.

[8] Lawrence W, Hays DM, Heyn R, Tefft M, Crist W, Beltangady M, Wharam M (1987). Lymphatic metastases with childhood rhabdomyosarcoma: a report from the Intergroup Rhabdomyosarcoma Study. Cancer.

[9] Julieron M, Robin YM, Penel N, Chevalier D (2013). Sarcomes de la tête et du cou. EMC Oto-rhino-laryngologie.

